# *﻿Thrixspermumtaeniophyllum* (Orchidaceae, Epidendroideae), a new species from southwest China, based on molecular and morphological evidence

**DOI:** 10.3897/phytokeys.230.104273

**Published:** 2023-08-07

**Authors:** Jun-Yi Zhang, Yue-Hong Cheng, Min Liao, Gui-Ying Liu, Pan-Yan Yang, Bo Xu, Hai He

**Affiliations:** 1 College of Life Sciences, Chongqing Normal University, Chongqing 401331, China Chengdu Institute of Biology, Chinese Academy of Sciences Chengdu China; 2 CAS Key Laboratory of Mountain Ecological Restoration and Bioresource Utilization & Ecological Restoration and Biodiversity Conservation Key Laboratory of Sichuan Province, Chengdu Institute of Biology, Chinese Academy of Sciences, Chengdu 610041, China Chongqing Normal University Chongqing China; 3 Wolong National Nature Reserve Administration Bureau, Wenchuan 623006, Sichuan, China Wolong National Nature Reserve Administration Bureau Wenchuan China; 4 University of Chinese Academy of Sciences, Beijing 10049, China University of Chinese Academy of Sciences Beijing China

**Keywords:** Epiphytic orchid, flora of Sichuan, phylogeny, systematic position, taxonomy

## Abstract

*Thrixspermumtaeniophyllum* is described as a new orchid species from Wenchuan County, Sichuan Province of southwest China. It is morphologically similar to *T.japonicum*, but it differs from the latter in having branched stems, slightly fleshy strap-shaped leaves, longer inflorescences with 3–6 flowers and a capitate gynandrium with a lip-shaped mouth opening. Its species status is also supported by molecular phylogenetic analyses, based on nuclear ribosome internal transcribed spacer (nrITS) and three chloroplast DNA fragments (*mat*K, *psb*A-*trn*H and *trn*L-F), which showed distinct systematic boundaries from the most morphologically similar *T.japonicum* and their morphological relatives *T.saruwatarii* and *T.pygmaeum*.

## ﻿Introduction

*Thrixspermum* Lour. (1790) is a genus of mostly medium-sized epiphytes and lithophytes in the family Orchidaceae Juss. and it is known to include ca. 160 species widely distributed from tropical and subtropical Asia to the islands of the western Pacific islands (Chen et al. 2009; Chase et al. 2015; Kumar et al. 2017). This genus is characterised by the persistent floral bracts, a three-lobed labellum and the four waxy subglobose pollinia grouped into two unequal masses in appearance (Loureiro 1790; Chen et al. 2009). It is also a congregation of elusive orchids with limited floral materials for morphological comparison due to their rather short flowering period (Govaerts et al. 2016). From accounts of ca. 17 species distributed in southern China, only one species, *T.japonicum* (Miq.) Reichenbach fils (1878), has been recorded from Sichuan Province (Song et al. 2009; Kumar et al. 2017; Zhou et al. 2021).

**Figure 1.**
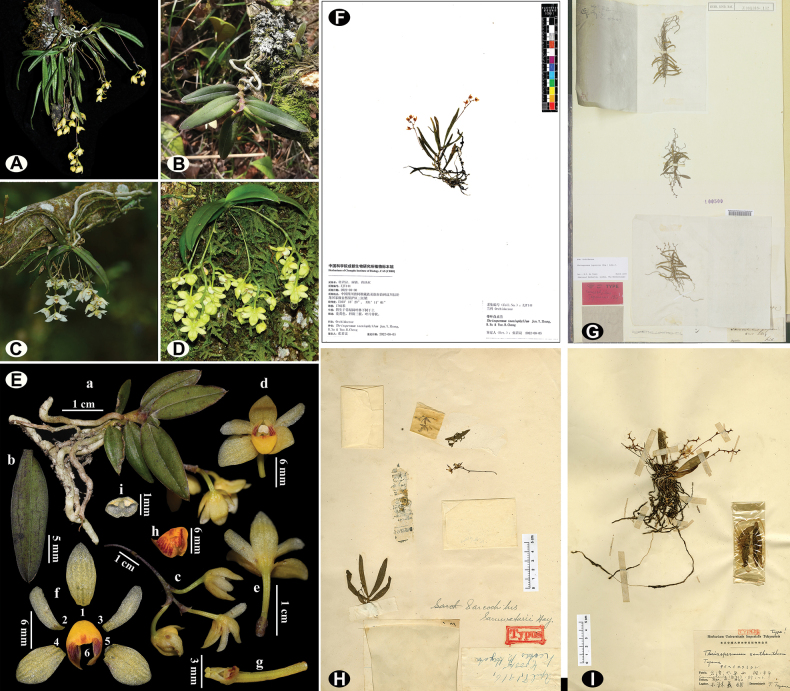
Comparison of four species of *Thrixspermum*. Living plant of *T.taeniophyllum* (**A**), *T.japonicum* (**B**), *T.saruwatarii* (**C**) and *T.pygmaeum* (**D**); Detailed colour photos of *T.japonicum* (**E: a** habit **b** leaves **c** inflorescence **d, e** flower in front and back view **f1** dorsal sepal **f2–3** petals **f4–5** sepals **f6, h** labellum **g** gynandrium and ovary **i** anther cap); Type specimens of *T.taeniophyllum* (**F**: holotype at CDBI), *T.japonicum***(G**: holotype at L) and *T.saruwatarii* (**H**: holotype at TI); and a representative specimen of *T.pygmaeum* (**I**: TI). [Images **A** and **E** photographed by Min Liao; image **B** photographed by Yue-Hong Cheng; image **C** cited from website (https://kevinyu589.blogspot.com/) image **D** cited from website (https://www.gbif.org/species/2846707); the image of the type specimen of *T.japonicum* was obtained from JSTOR and those of *T.saruwatarii* and *T.pygmaeum* were cited from available from Plants of Taiwan (https://tai2.ntu.edu.tw/search/2)].

As part of a continuous inventory of orchids from Sichuan, China, we have conducted continuous field explorations in the Wenchuan section of the Giant Panda National Park. During a field trip in March 2022, we encountered an interesting epiphytic orchid in Wolong National Nature Reserve (Wenchuan County, Sichuan Province, China), that we had initially identified it as *Thrixspermumjaponicum*, based on its pendulous inflorescence, golden-yellow flowers and orange-red striped lateral lobes of the labellum. However, upon a critical morphological observation and comparison with available specimens including the type materials of two of the three [we were unsuccessful to trace the type specimen(s) of *T.pygmaeum* (King & Pantl.) Holttum (1960)] morphologically related species (Fig. 1A–I), for example, *T.japonicum* (*P. F. V. Siebold, s.n.*, L) and *T.saruwatarii* (Hayata) Schlechter (1919) (*T01201*, TI), we assumed it to be a new species of *Thrixspermum* that we are now describing hereafter.

## ﻿Materials and methods

### ﻿Morphological observations

Morphological information (including the colour, size and shape of the roots, stems, leaves, flowers and floral parts, details see Table 1) of this new species was obtained from observations and measurements of eight living plants in the field and four dried herbarium specimens (voucher information: *Jun-Yi Zhang, Min Liao & Yue-Hong Cheng ZJY144*; *Jun-Yi Zhang & Yue-Hong Cheng ZJY189*; *Jun-Yi Zhang & Yue-Hong Cheng ZJY191*; *Jun-Yi Zhang & Yue-Hong Cheng ZJY192*) deposited at CDBI (acronym of herbarium follows Thiers 2023). Voucher information for the four specimens used for morphological observations and their collection location are detailed in the taxonomic treatment. The terminology in Beentje (2012) was followed for the description.

**Table 1.** Morphological comparison amongst *Thrixspermumtaeniophyllum*, *T.japonicum*, *T.saruwatarii* and *T.pygmaeum*. Characters of the last three species are modified from the respective protologues (Miquel 1866; King and Pantling 1898; Hayata 1916) and Flora of China (Chen et al. 2009).

Character	*T.taeniophyllum*	*T.japonicum*	*T.saruwatarii*	*T.pygmaeum*
Stems	**4–8 cm long, often branched, internodes 5–8 mm**	3–13 cm long, unbranched, internodes 3–5 mm	shorter than 2 cm, unbranched, internodes ≤ 1 mm	shorter than 3 cm, unbranched, internodes ≤ 1 mm
Leaves	dichotomously alternate, **slightly fleshy**, **strap-shaped**, **5–7 × 0.5–1 cm**	dichotomously alternate, thinly leathery, oblong, 2–4 × 0.5–0.7 cm	nearly basal, narrowly oblong or linear-oblanceolate, 4–8 × 0.5–2 cm	nearly basal, elliptic to linear-oblong, rarely falcate, 2–8 × 0.7–1.5 cm
Inflorescences	**6–12 cm long, with 3–6 flowers**	3–5 cm long, with 2–3 flowers	longer than 8 cm, with 1–4 flowers	2–4 cm long, with 2–4 flowers
Floral bracts	broadly ovate-triangular, ca. 4 mm	broadly ovate-triangular, ca. 2.5 mm	ovate-triangular, 2–3 mm	ovate, 2–3 mm
Dorsal sepal	elliptic, 5–7 × 3.5–4.5 mm	oblong, 5–7 × 2.5–3 mm	oblong, 7–8 × 3–5 mm	elliptic, 6–8 × 4–5 mm
Lateral sepals	elliptic, 5–7 × 3.5–4.5 mm	ovate-lanceolate, 5–7 × 2.5–3 mm	slightly oblique, 7–8 × 3–5 mm	obliquely ovate, 6–7 × 4–5 mm
Petals	narrowly elliptic, 4.5–6 × 2–3 mm	narrowly oblong, 5–6 × 1.5–2 mm	linear, falcate, 5–6 × ca. 2 mm	oblong-spatulate, 6–7 × 2–3 mm
Lateral lobes of labellum	erect, nearly oblong, ca. 2.5 mm	narrowly ovate-oblong, ca. 2.5 mm	erect, falcate, ca. 3 mm	erect, oblong, falcate, 6–7 mm
Mid-lobe of labellum	fleshy, very small, teeth triangular	fleshy, very small, semi-orbicular	fleshy, very small, triangular	fleshy, small, semi-orbicular
Lip disc	without a callus, slightly depressed, with red purple or golden yellow hairs	with a callus, slightly depressed, densely tomentose	with a callus, where a tuft of brownish-yellowish hairs arises	without a callus, slightly depressed, with a tuft of purple hairs
Gynandrium	**Capitate, mouth opening lip-shaped**	conical, mouth opening triangular	cylindrical, mouth opening semi-lunar	cylindrical, mouth opening semi-lunar

### ﻿DNA extraction and sequencing

The sequences of four individuals of this new species from two different areas (Wolong and Gengda towns) in Wenchuan County and two individuals of *T.japonicum* (vouchers *Jun-Yi Zhang & Yue-Hong Cheng ZJY187* and *Jun-Yi Zhang & Yue-Hong Cheng ZJY188*, deposited at CDBI) were newly obtained in this study with the following protocols. Total DNA was extracted from silica-gel dried leaves via a Plant DNA Isolation Kit (Cat.No.DE-06111, Foregene, Chengdu, China). The sequences were amplified by means of the primers (Table 2) used in previous studies of *Thrixspermum* (Li et al. 2014; Zou et al. 2015). The PCR programme consisted of an initial 4 min preheating stage at 98 °C, followed by 35 cycles of 30 s at 98 °C (denaturation), 30 s at 48–56 °C (annealing) and 60–100 s at 68 °C (extension), followed by a final 8 min extension at 68 °C. The PCR products were sent to TSINGKE Biotech (Chengdu, China) for sequencing. The returned sequences were edited via Sequencher v.4.1.4 (Gene Codes, Ann Arbor, Michigan, USA) and checked manually and then deposited in the GenBank with the following accession numbers: nrITS (OP348891, OQ608783, OR054231, OR054232, OR054229, OR054230), *mat*K (OP373116, OQ626557, OR062235, OR062236, OR062233, OR062234), *psb*A-*trn*H (OP373121, OQ626556, OR062240, OR062241, OR062238, OR062239) and *trn*L-F (OR184926, OR184927, OR062245, OR062246, OR062243, OR062244), respectively.

**Table 2.** Information of DNA markers used in this study for *Thrixspermum*.

DNA markers	Length (bp)	Variable sites (bp)	Primer sequence (5’to3’)	Origin
nrITS	675	239	ACGAATTCATGGTCCGGTGAAGTGTTCG	Sun et al. (1994)
			GAATTCCCCGGTTCGCTCGCCGTTAC	Sun et al. (1994)
*psb*A-*trn*H	748	44	GTTATGCATGAACGTAATGCTC	Sang et al. (1997)
			CGCGCATGGTGGATTCACAAATC	Sang et al. (1997)
*mat*K	881	122	CGATCTATTCATTCAATATTTC	Sun et al. (1994)
			TCTAGCACACGAAAGTCGA	Sun et al. (1994)
*trn*L-F	908	94	AAAATCGTGAGGGTTCAAGTC	Sang et al. (1997)
			GATTTGAACTGGTGACACGAG	Sang et al. (1997)

### ﻿Phylogenetic analyses

A total of 54 accessions representing 44 taxa were incorporated in the phylogenetic analysis, including *Phalaenopsismarriottiana* (Rchb. f.) Kocyan & Schuiteman (2014) as outgroup. The ingroup includes 36 entities of *Thrixspermum* representing 26 species and 17 taxa belonging to six related genera in Aeridinae (Orchidaceae, Epidendroideae) following the two previous studies of Li et al. (2014) and Zou et al. (2015). The detailed information concerning the sampled taxa, voucher specimens and GenBank accession numbers (including the sequences retrieved from GenBank) used for the phylogenetic analyses are summarised in Appendix 1. The nrITS, *mat*K, *psb*A-*trn*H and *trn*L-F matrices contain 40, 22, 13 and 13 taxa, respectively (Appendix 1). All sequences were aligned using MAFFT v.7.475 (Katoh and Standley 2013) with default parameters. The incongruence length difference test (ILD) was used to quantify the conflicts between nuclear DNA (nrITS) and plastid DNA (*mat*K, *psb*A-*trn*H, *trn*L-F) data in PAUP v.4.0a169 (Darlu and Lecointre 2002; Swofford 2002). The ILD Test (P = 0.11) indicated that nrITS and plastid datasets were suitable for combined analysis in *Thrixspermum* and, thus, the results are based on the combined data of nrITS and three plastid markers. The nucleotide substitution models for these data matrices were estimated using the software jModelTest v.2.1.6 (Posada 2008) and the best fit models were selected using the corrected Akaike Information Criterion (AICc). Bayesian Inference (BI) and Maximum Likelihood (ML) analyses were performed to infer the phylogenetic relationships within the combined dataset. The BI analysis was conducted using MrBayes v.3.2.7a (Ronquist and Huelsenbeck 2003), with two separate Markov Chain Monte Carlo (MCMC) chains (1,000,000 generations and sampled every 1,000 generations). The first 25% of the trees were discarded as burn-in and the remaining trees were used to generate a majority-rule consensus tree. The ML analysis was performed using IQ-TREE v.1.4.2 (Nguyen et al. 2014) with branch support estimated using 2,000 replicates of both SH-like approximate likelihood-ratio test (SH-aLRT) (Guindon et al. 2010) and the ultrafast bootstrapping algorithm (UFboot) (Minh et al. 2013).

## ﻿Results

**Figure 2.**
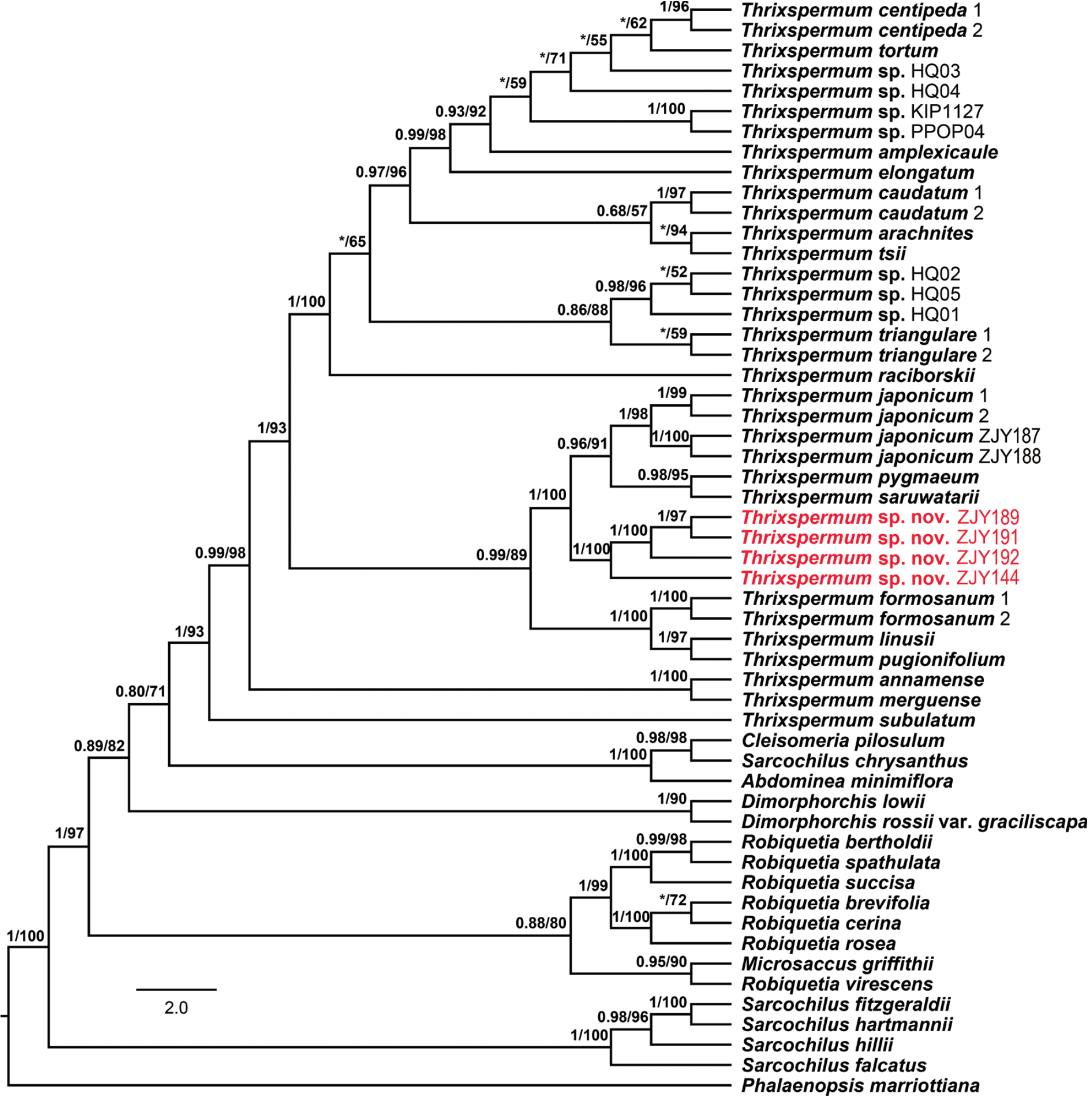
Maximum Likelihood tree of 36 entities of *Thrixspermum* reconstructed, based on combined nuclear and plastid dataset. Numbers before slash indicate Bayesian posterior probabilities and numbers after slash indicate ML bootstrap supports for major lineages. Asterisk (*) indicates that a node is not supported in the analysis. The four individuals of the inferred new species are highlighted in red.

The aligned nrITS matrix of 48 accessions (40 taxa) was 675 nucleotides in length with 239 variable sites and plastid matrix of 30 accessions (23 taxa) was 2537 nucleotides in length with 260 variable sites, of which 881 bp for *mat*K (29 accessions, 22 taxa, 122 variable sites), 748 bp for *psb*A-*trn*H (20 accessions, 13 taxa, 44 variable sites) and 908 bp for *trn*L-F (19 accessions, 13 taxa, 94 variable sites), respectively (Table 2). Phylogenetic analyses indicated that the 26 included taxa of *Thrixspermum* formed a well-supported monophyletic group (Fig. 2). Four individuals of the inferred new species from the two sites in Wenchuan County were resolved as a strongly-supported monophyletic lineage (Fig. 2; BI/ML = 1/100%), which further clustered with *T.japonicum*, *T.saruwatarii* and *T.pygmaeum* into a subclade (Fig. 2; BI/ML = 1/100%). These four species also showed certain morphological similarities (referring to Fig. 1 and Table 1). It is noted that *T.japonicum* is the most related species to the novelty by sharing with the new species pendulous inflorescence, inside brownish-striped lateral lobes and densely hairy small triangular mid-lobe of labellum (Fig. 1B, E). Besides, the following morphological diagnosis, their molecular boundary is clearly shown by the positions of their respective individuals as well (Fig. 2).

### ﻿Taxonomic treatment

#### ﻿ Thrixspermum taeniophyllum

Taxon classificationPlantaeAsparagalesOrchidaceae

Jun Y.Zhang, H.He & Yue H.Cheng sp. nov.

EECE991C-23A6-5D43-BDF8-4A9F83D6740F

urn:lsid:ipni.org:names:77324993-1

Figs 1A , 3

##### Type.

China. Sichuan Province, Wenchuan County, Wolong Town, in coniferous and broadleaf mixed forest, on tree trunks, elev. ca. 1762 m, in flower, 30 March 2022, *Jun-Yi Zhang, Min Liao & Yue-Hong Cheng ZJY144* (holotype CDBI!).

**Figure 3.**
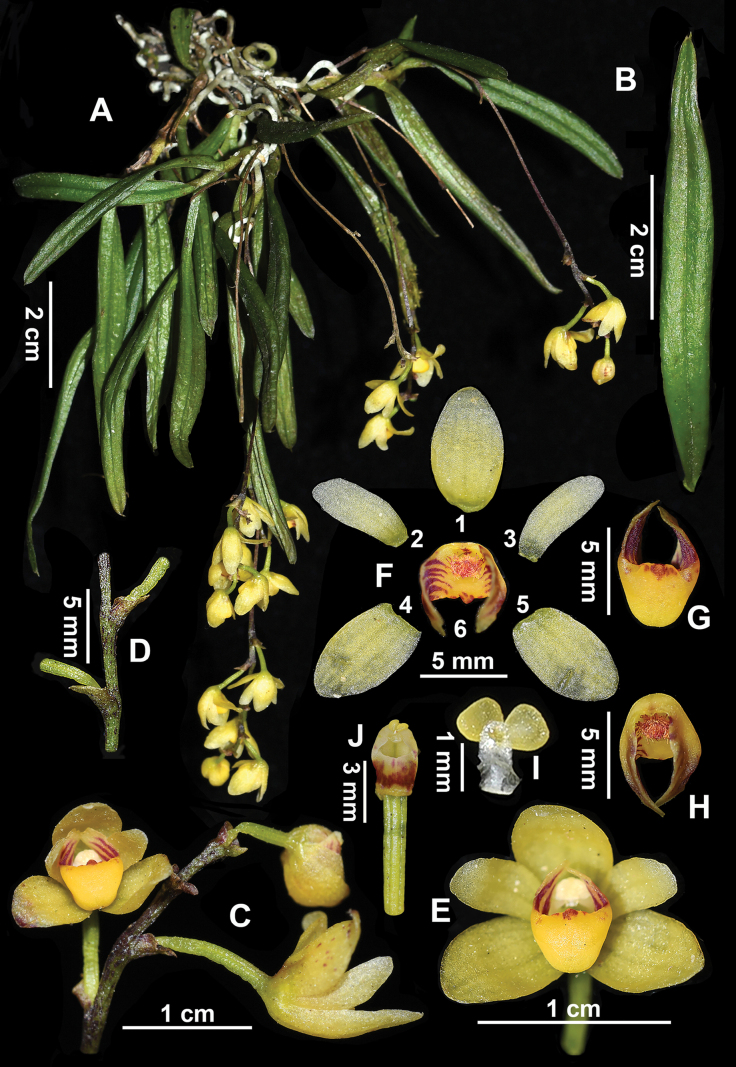
*Thrixspermumtaeniophyllum***A** flowering plant **B** leaves **C** inflorescence **D** part of the rachis showing persistent bracts **E** flower in front view **F** dissection of a flower (F1: dorsal sepal; F2–F3: petals; F4–F5: sepals; F6: labellum) **G, H** labellum **I** pollinia **J** gynandrium and ovary.

##### Diagnosis.

It is most similar to *Thrixspermumjaponicum* in morphology, but it differs from the latter by its often-branched stems (vs. unbranched stems), slightly fleshy strap-shaped leaves 5–7 cm long (vs. thinly leathery oblong leaves 2–4 cm long), longer inflorescences with 3–6 flowers (vs. shorter inflorescences with 2–3 flowers) and its capitate gynandrium with a lip-shaped mouth opening (vs. conical gynandrium with a triangular mouth opening). It also resembles *T.saruwatarii* and *T.pygmaeum* and morphological comparison amongst the four species is visualised in Fig. 1 and summarised in Table 1.

##### Description.

**Epiphytic** on tree trunks. **Roots** vermiform and slender. **Stems** ascending or pendulous especially when in flowering, 4–8 cm long, ca. 1.5 mm thick, often branched, internodes 5–8 mm apart. **Leaves** green, dichotomously alternate, slightly fleshy, strap-shaped, 5–7 × 0.5–1 cm, apex acute and bifid with two unequally mucronate tips. **Inflorescence** arising from basal stem laterally or opposite to leaves, usually pendulous, 6–12 cm long; rachis slightly flexuous and slightly thickened distally, 4–6 cm long, laxly 3–6 flowered; bracts spirally arranged, obliquely patent, ca. 3 mm long, broadly ovate-triangular, apex acute. **Flowers** initially white and later turning creamy yellow, blossoming almost simultaneously; dorsal and lateral sepals similar, elliptic, 5–7 × 3.5–4.5 mm, apex obtuse, with obscure 3 veins; petals narrowly elliptic, smaller than sepals, 4.5–6 × 2–3 mm, apex obtuse, with obscure 1 or 2 veins; labellum small, three-lobed, base shallowly saccate; lateral lobes erect, triangularly oblong, ca. 2.5 mm, apex rounded or ± notched, inner surface with many purplish-red stripes; mid-lobe fleshy, reddish-brown, very small, apex ended with triangular teeth; disc concave, inside base densely covered with red purple or golden yellow hairs; gynandrium capitate, with a lip-shaped mouth opening; ca. 2.5 mm high; column foot ca. 1.2 mm, with one joint at the junction with the labellum; pollinia 4 grouped into two nearly similar masses, ca. 0.8 × 0.7 mm, yellow, full and obovately spherical. **Fruits** unseen.

##### Distribution and habitat.

*Thrixspermumtaeniophyllum* was found in Wenchuan County, Sichuan Province, southwest China. It is epiphytic on trees in coniferous and broadleaf mixed forest at an elevational range between 1260 and 1770 m.

##### Phenology.

Flowering in March and April.

##### Etymology.

The specific epithet *taeniophyllum* is a compound adjective referring to the shape of leaves of this new species. A Chinese name, dai ye bai dian lan (带叶白点兰), is also suggested, based on the its leaf feature.

##### Additional specimens examined.

China. Sichuan Province, Wenchuan County, Wolong Town, evergreen broad-leaved forest, on tree trunks, elev. ca. 1769 m, in flower, 1 May 2023, *Jun-Yi Zhang & Yue-Hong Cheng ZJY189* (CDBI!); Sichuan Province, Wenchuan County, Gengda Town, evergreen broad-leaved forest, on tree trunks, elev. ca. 1508 m, in flower, 2 May 2023, *Jun-Yi Zhang & Yue-Hong Cheng ZJY191* (CDBI!); Sichuan Province, Wenchuan County, Gengda Town, evergreen broad-leaved forest, on tree trunks, elev. ca. 1520 m, in flower, 2 May 2023, *Jun-Yi Zhang & Yue-Hong Cheng ZJY192* (CDBI!).

##### Examined specimens of Thrixspermumjaponicum.

Japan. *P. F. V. Siebold, s.n.* (holotype L; It is not clear whether duplicates exist); China. Sichuan, Emeishan, elev. ca. 990 m, 12 July 1980, *K. Y. Lang, B. C. Gao et al. 044* (PE); Baoxing, elev. ca. 1800 m, 2 April 1983, *D. Y. Peng 47469* (CDBI!); Beichuan, elev. ca. 1640 m, 1 August 1984, *C. L. Tang et al. 284* (CDBI!). Chongqing: Nanchuan, elev. ca. 900 m, 2 November 1983, *Z. Y. Liu 4669* (IMC); Nanchuan, elev. ca. 850 m, 30 May 1984, *Z. Y. Liu 5219* (PE).

##### Examined specimens of Thrixspermumsaruwatarii.

China. Taiwan, Alishan, 8 April 1916, *B. Hayata, s.n.* (holotype TI); Kaohsiung, 20 October 1934, *S. Sasaki, s.n.* (TAI). Yunnan, Gongshan, elev. ca. 1702 m, 20 May 2007, *X. H. Jin 9001* (PE). Hunan, Ningyuan, elev. ca. 340 m, 8 May 2011, *X. L. Yu 11050801* (CSFI). Guangdong, Dapu, elev. ca. 700 m, 9 June 1957, *L. Deng 4953* (IBSC).

##### Examined specimens of Thrixspermumpygmaeum.

China. Taiwan, Taipingshan, 3 April 1940, *Y. Kobayashi, s.n.* (TI); Taitung, 22 March 1932, *S. Sasaki, s.n.* (TAI); Miaoli, 13 November 1972, *C. C. Hsu 12600* (TAI); Ilan, 10 March 2013, *C. C. Hsu, s.n.* (TAI).

### ﻿Key to the four related species of *Thrixspermum*

**Table:** 

1a	Stems shorter than 3 cm, internodes ≤ 1 mm; leaves nearly basal	**2**
2a	Inflorescences longer than 8 cm; lip disc with a callus, where a tuft of brownish-yellowish hairs arises	***T.saruwatarii***
2b	Inflorescences 2–4 cm long; lip disc without a callus, slightly depressed, with a tuft of purple hairs	***T.pygmaeum***
1b	Stems longer than 3 cm, internodes ≥ 3 mm; leaves distichous alternate	**3**
3a	Stems branched; leaves strap-shaped, 5–7 × 0.5–1 cm; inflorescences 6–12 cm long with 3–6 flowers and a capitate gynandrium with a lip-shaped mouth opening	***T.taeniophyllum***
3b	Stems unbranched; leaves oblong, 2–4 × 0.5–0.7 cm; inflorescences 3–5 cm long with 2–3 flowers and a conical gynandrium with a triangular mouth opening	***T.japonicum***

## Supplementary Material

XML Treatment for Thrixspermum taeniophyllum

## ﻿Additional information

### Author ORCIDs

Jun-Yi Zhang https://orcid.org/0000-0001-8325-5954

Yue-Hong Cheng https://orcid.org/0000-0001-6263-0989

Min Liao https://orcid.org/0000-0001-6777-8579

Gui-Ying Liu https://orcid.org/0000-0003-4905-2429

Pan-Yan Yang https://orcid.org/0009-0004-0722-8953

Bo Xu https://orcid.org/0000-0003-3507-9321

Hai He https://orcid.org/0000-0001-5362-9177

### Data availability

All of the data that support the findings of this study are available in the main text or Appendix 1.

## ﻿Appendix 1

**Table A1.** The GenBank accession numbers for DNA sequences used in this study.

Taxa	Voucher	nrITS	*psb*A-*trn*H	*mat*K	*trn*L-F
*Abdomineaminimiflora*	B200107222	AB217524	—	—	—
*Cleisomeriapilosulum*	TBG140482	AB217542	—	AB217718	—
*Dimorphorchislowii*	TBG118871	AB217548	—	AB217724	—
Dimorphorchisrossiivar.graciliscapa	Botanical Garden Heidelberg 122351	EF670358	—	EF655807	—
*Microsaccusgriffithii*	KFBG2673	KY966620	—	—	—
*Phalaenopsismarriottiana*	Z. J. Liu 8743	KX579760	KX579764	KX579762	KX579766
*Robiquetiabertholdii*	Chase 17866	—	—	FR832824	—
*Robiquetiabrevifolia*	WAMP_ORCH-13	MT505292	—	—	—
*Robiquetiacerina*	Carlsward 210 (SEL)	DQ091701	—	—	—
*Robiquetiarosea*	WAMP_ORCH-14	MT505293	—	—	—
*Robiquetiaspathulata*	Z. J. Liu 6691	—	KJ733523	KF421855	KJ733680
*Robiquetiasuccisa*	Z. J. Liu 5248	KJ733444	KJ733524	KJ733601	KJ733681
*Robiquetiavirescens*	WAMP_ORCH-15	MT505294	—	—	—
*Sarcochiluschrysanthus*	TBG145831	AB217582	—	AB217757	—
*Sarcochilusfalcatus*	A. Perkins 14	AF321600	—	—	—
*Sarcochilusfitzgeraldii*	Carlsward 231 (FLAS)	DQ091728	—	—	—
*Sarcochilushartmannii*	TBG145793	AB217581	—	AB217758	—
*Sarcochilushillii*	Perkins 15	AF321601	—	—	—
*Thrixspermumamplexicaule*	Z. J. Liu 4971	KF545882	KF545871	KF545892	KF545904
*Thrixspermumannamense*	Z. J. Liu 4972	KF545883	KF545872	KF545893	KF545905
*Thrixspermumarachnites*	Heidelberg BG 104401	—	—	EF065572	—
*Thrixspermumcaudatum* 1	KIP0690	KX679330	—	—	—
*Thrixspermumcaudatum* 2	KIP0352	KX679331	—	—	—
*Thrixspermumcentipeda* 1	KIP06	KX679341	—	—	—
*Thrixspermumcentipeda* 2	KFBG3306AL	KY966675	KJ733536	KJ733621	KJ733691
*Thrixspermumelongatum*	Carlsward 170 (SEL)	DQ091674	—	—	—
*Thrixspermumformosanum* 1	OT00257	—	KJ733540	KJ733620	KJ733695
*Thrixspermumformosanum* 2	Yue-Hong Cheng 321	OR054228	OR062237	OR062232	OR062242
*Thrixspermumjaponicum* 1	PDBK2015-1270	—	KX871234	KX871234	KX871234
*Thrixspermumjaponicum* 2	S. A. Choi 784	KT338782	KF262223	KF262105	—
*Thrixspermumjaponicum* ZJY187	Jun-Yi Zhang & Yue-Hong Cheng ZJY187	OR054229	OR062238	OR062233	OR062243
*Thrixspermumjaponicum* ZJY188	Jun-Yi Zhang & Yue-Hong Cheng ZJY188	OR054230	OR062239	OR062234	OR062244
*Thrixspermumlinusii*	KIP1150	KX679333	—	—	—
*Thrixspermummerguense*	KIP1094	KX679334	—	—	—
*Thrixspermumpugionifolium*	WAMP_ORCH-17	MT505296	—	MT966905	—
*Thrixspermumpygmaeum*	OT00263	KJ733457	KJ733537	KJ733613	KJ733692
*Thrixspermumraciborskii*	AD7LN53	—	MF348752	MF349945	—
*Thrixspermumsaruwatarii*	Z. J. Liu 3905	KJ733458	KJ733538	KJ733614	KJ733693
*Thrixspermum* sp. PPOP04	PPOP04	KX679342	—	—	—
*Thrixspermum* sp. HQ03	HQ03	KX679338	—	—	—
*Thrixspermum* sp. HQ04	HQ04	KX679339	—	—	—
*Thrixspermum* sp. KIP1127	KIP1127	KX679335	—	—	—
*Thrixspermum* sp. HQ02	HQ02	KX679337	—	—	—
*Thrixspermum* sp. HQ05	HQ05	KX679340	—	—	—
*Thrixspermum* sp. HQ01	HQ01	KX679336	—	—	—
*Thrixspermumsubulatum*	TBG113211	AB217592	—	AB217768	—
*Thrixspermumtortum*	KIP0185	KX679347	—	—	—
*Thrixspermumtriangulare*	HQ06	KX679348	—	—	—
*Thrixspermumtriangulare*	980162 (L)	EF670367	—	—	EF670412
*Thrixspermumtsii*	Z. J. Liu 3264	KJ733459	KJ733539	KJ733615	KJ733694
*Thrixspermumtaeniophyllum* ZJY144	Jun-Yi Zhang, Min Liao & Yue-Hong Cheng ZJY144	OP348891	OP373121	OP373116	OR184926
*Thrixspermumtaeniophyllum* ZJY189	Jun-Yi Zhang, Min Liao & Yue-Hong Cheng ZJY189	OQ608783	OQ626556	OQ626557	OR184927
*Thrixspermumtaeniophyllum* ZJY191	Jun-Yi Zhang, Min Liao & Yue-Hong Cheng ZJY191	OR054231	OR062240	OR062235	OR062245
*Thrixspermumtaeniophyllum* ZJY192	Jun-Yi Zhang, Min Liao & Yue-Hong Cheng ZJY192	OR054232	OR062241	OR062236	OR062246

## References

[B1] Beentje H (2012) The Kew Plant Glossary, an illustrated dictionary of plant terms. Kew Publishing, London, 164 pp.

[B2] Chase MW Cameron KM Freudenstein JV Pridgeon AM Salazar G Berg CVD Schuiteman A (2015) An updated classification of Orchidaceae. Botanical Journal of the Linnean Society 177(2): 151–174. 10.1111/boj.12234

[B3] Chen SC Tsi ZH Wood JJ (2009) *Thrixspermum* Lour. In: Wu ZY Raven PH Hong DY (Eds) Flora of China, vol.19 (Orchidaceae). Missouri Botanical Garden Press, St. Louis and Science Press, Beijing, 336–342.

[B4] Darlu P Lecointre G (2002) When does the incongruence length difference test fail? Molecular Biology and Evolution 19(4): 432–437. 10.1093/oxfordjournals.molbev.a004098 11919284

[B5] Govaerts R Campacci MA Baptista DH Baptista PJ George A Kreutz K Wood JJ (2016) World Checklist of Orchidaceae. The Board of Trustees of the Royal Botanic Gardens, Kew. http://apps.kew.org/wcsp/

[B6] Guindon S Dufayard JF Lefort V Anisimova M Hordijk W Gascuel O (2010) New algorithms and methods to estimate Maximum-Likelihood phylogenies: Assessing the performance of PhyML 3.0. Systematic Biology 59(3): 307–321. 10.1093/sysbio/syq010 20525638

[B7] Hayata B (1916) Icones Plantarum Formosarum nec non et contributiones ad floram Formosanam, vol. 6. Governmnet of Formosa, Taihoku, 168 pp.

[B8] Holttum RE (1960) The genera *Sarcochilus* R. Br. and *Pteroceras* Hassk. (Orchidaceae) with notes on other genera which have been included in *Sarcochilus*. Kew Bulletin 14(2): 263–276. 10.2307/4114807

[B9] Katoh K Standley DM (2013) MAFFT multiple sequence alignment software version 7: Improvements in performance and usability. Molecular Biology and Evolution 30(4): 772–780. 10.1093/molbev/mst010 23329690 PMC3603318

[B10] King G Pantling R (1898) Orchids of the Sikkim-Himalaya. Annals of the Royal Botanic Garden, Calcutta 8: 207.

[B11] Kocyan A Schuiteman A (2014) New combinations in Aeridinae (Orchidaceae). Phytotaxa 161(1): 61–85. 10.11646/phytotaxa.161.1.3

[B12] Kumar V Verma D Rao AN (2017) *Thrixspermumindicum* (Orchidaceae), a new species from Northeast India. Phytotaxa 292(1): 79–84. 10.11646/phytotaxa.292.1.8

[B13] Li MH Zhang GQ Liu ZJ Lan SR (2014) Revision of *Hygrochilus* (Orchidaceae: Epidendroideae: Aeridinae) and a molecular phylogenetic analysis. Phytotaxa 159(4): 256–268. 10.11646/phytotaxa.159.4.2

[B14] Loureiro J (1790) Flora Cochinchinensis vols. 1–2. Typis et Expensis Academicis, Lisbon, 744 pp.

[B15] Minh BQ Nguyen MAT von-Haeseler A (2013) Ultrafast approximation for phylogenetic bootstrap. Molecular Biology and Evolution 30(5): 1188–1195. 10.1093/molbev/mst024 23418397 PMC3670741

[B16] Miquel FAW (1866) Prolusio florae laponicae. Annales Musei Botanici Lugduno-Batavi 2: 69–212, 257–300.

[B17] Nguyen LT Schmidt HA Von-Haeseler A Minh BQ (2014) IQ-TREE: A fast and effective stochastic algorithm for estimating Maximum-Likelihood phylogenies. Molecular Biology and Evolution 32(1): 268–274. 10.1093/molbev/msu300 25371430 PMC4271533

[B18] Posada D (2008) jModelTest: Phylogenetic model averaging. Molecular Biology and Evolution 25(7): 1253–1256. 10.1093/molbev/msn083 18397919

[B19] Reichenbach HG (1878) Ad Orchidographiam Japonicam Symbolae. Botanische Zeitung (Berlin) 36: 74–76.

[B20] Ronquist F Huelsenbeck JP (2003) MrBayes 3: Bayesian phylogenetic inference under mixed models. Bioinformatics (Oxford, England) 19(12): 1572–1574. 10.1093/bioinformatics/btg180 12912839

[B21] Sang T Crawford D Stuessy T (1997) Chloroplast DNA phylogeny, reticulate evolution, and biogeography of *Paeonia* (Paeoniaceae). American Journal of Botany 84(8): 1120–1136. 10.2307/2446155 21708667

[B22] Schlechter FRR (1919) Orchideologiae Sino-Japonicae prodromus. Eine kritische Besprechtung der Orchideen Ost-Asien. Repertorium Specierum Novarum Regni Vegetabilis. Beihefte 4: 1–319.

[B23] Song XQ Meng QW Wing YT Luo YB (2009) *Thrixspermumodoratum* (Orchidaceae), a new species from Hainan Island, China. Annales Botanici Fennici 46(6): 595–598. 10.5735/085.046.0617

[B24] Sun Y Skinner D Liang G Hulbert S (1994) Phylogenetic analysis of *Sorghum* and related taxa using internal transcribed spacers of nuclear ribosomal DNA. Theoretical and Applied Genetics 89(1): 26–32. 10.1007/BF00226978 24177765

[B25] Swofford DL (2002) PAUP*: phylogenetic analysis using parsimony (*and other methods), version 4.0 a169. Sinauer Associates, Sunderland.

[B26] Thiers B (2023) Index Herbariorum: a global directory of public herbaria and associated staff. New York Botanical Garden’s Virtual Herbarium. http://sweetgum.nybg.org/science/ih

[B27] Zhou ZH Shi RH Zhang Y Xing XK Jin XH (2021) Orchid conservation in China from 2000 to 2020: Achievements and perspectives. Plant Diversity 43(5): 343–349. 10.1016/j.pld.2021.06.003 34816060 PMC8591184

[B28] Zou YJ Chen ZJ Kondo K Funamoto T Wen J Zhou SL (2015) A molecular phylogeny of Aeridinae (Orchidaceae: Epidendroideae) inferred from multiple nuclear and chloroplast regions. Molecular Phylogenetics and Evolution 85: 247–254. 10.1016/j.ympev.2015.02.014 25725112

